# A Manual of Bacteriology

**Published:** 1899-06

**Authors:** 


A Manual of Bacteriology. By Herbert U. Williams, Professor of Pathology and Bacteriology, Medical Department, University of Buffalo. 8vo, pp. 263. With 78 illustrations. Philadelphia: P. Blakiston's Son & Co. 1898.

The many friends of Dr. Williams will be pleased with the success which his manual of bacteriology has attained. Although designed primarily for the use of the beginner in the laboratory, it also gives a concise summary of the facts in bacteriology most important to physicians. Of the smaller works on bacteriology Dr. Williams's manual is decidedly the best and is to be commended and recommended. Following an introduction in which the author indulges in plain talk on bacteria, the book is divided into four parts. Part I. is devoted to bacteriological technique, instrument, staining methods; sterilisation; culture media; cultivation of bacteria; inoculation of animals and collection of material.

On page 29 is given a very valuable table, showing a list of the important bacteria that are stained by Gram's method, and a list of bacteria not stained by this method.

Part II. is devoted to a study of the bacteria themselves, their classification, product and growth, systematic study of a species of bacteria, distribution of bacteria in air, water, soil, in the healthy tissues of the body and in disease. A very able chapter on disinfectants and antiseptics is contributed by Dr. Thomas B. Carpenter, of Buffalo, and another equally as valuable and interesting on preparation of instruments, ligatures, dressings, etc., for surgical purposes by Dr. Chauncey P. Smith, also of Buffalo. These two chapters aid the book materially and are of interest and great profit to the busy practitioner, who has not the time to search through his chemistry for information regarding the different antiseptic agents.

Part III. is devoted to the nonpathogenic bacteria, and Part IV. to the pathogenic bacteria. The illustrations, numbering 78 in number, help materially in elucidating the text. On the whole, Dr. Williams's efforts are highly successful. W. C. K.

